# Four Dissemination Pathways for a Social Media–Based Breastfeeding Campaign: Evaluation of the Impact on Key Performance Indicators

**DOI:** 10.2196/14589

**Published:** 2019-09-26

**Authors:** Kassandra Harding, Rafael Pérez-Escamilla, Grace Carroll, Richmond Aryeetey, Opeyemi Lasisi

**Affiliations:** 1 Yale School of Public Health New Haven, CT United States; 2 School of Public Health University of Ghana Legon, Accra Ghana

**Keywords:** social media, health communication, breastfeeding, dissemination, Ghana

## Abstract

**Background:**

Social media utilization is on the rise globally, and the potential of social media for health behavior campaigns is widely recognized. However, as the landscape of social media evolves, so do techniques used to optimize campaign dissemination.

**Objective:**

The primary aim of this study was to evaluate the impact of 4 material dissemination paths for a breastfeeding social media marketing campaign in Ghana on exposure and engagement with campaign material.

**Methods:**

Campaign materials (n=60) were posted to a Facebook and Twitter campaign page over 12 weeks (ie, baseline). The top 40 performing materials were randomized to 1 of 4 redissemination arms (control simply posted on each platform, key influencers, random influencers, and paid advertisements). Key performance indicator data (ie, exposure and engagement) were extracted from both Facebook and Twitter 2 days after the material was posted. A difference-in-difference model was used to examine the impact of the dissemination paths on performance.

**Results:**

At baseline, campaign materials received an average (SD) exposure of 1178 (670) on Facebook and 1071 (905) on Twitter (n=60). On Facebook, materials posted with paid advertisements had significantly higher exposure and engagement compared with the control arm (*P*<.001), and performance of materials shared by either type of influencer did not differ significantly from the control arm. No differences in Twitter performance were detected across arms.

**Conclusions:**

Paid advertisements are an effective mechanism to increase exposure and engagement of campaign posts on Facebook, which was achieved at a low cost.

## Introduction

During social media’s early days in 2005, a mere 5% of Americans utilized it, while more recently in 2018, it was used by 67% and 49% of adults in advanced and emerging economies, respectively [1]. Over the past 14 years, the social media options have expanded to include a variety of platforms for a variety of purposes, ranging from Facebook’s focus on networking and relationships, to YouTube’s emphasis on information sharing, to Twitter’s emphasis on conversation [2]. Collectively, social media represents online platforms in the era of Web 2.0, on which user-generated content can be created or exchanged [3]. These platforms are heavily trafficked by their users. A survey conducted in the United States found that 74% of Facebook users visit the platform at least daily and 51% visited the platform multiple times a day [4]. Among teens, social media is a ubiquitous part of life, with 89% of teens participating in a Pew Research Survey reporting that they were online *almost constantly* (45%) or several times a day (44%) [5]. Given the time spent on social media and the abundance of information users are exposed to, social marketers need to utilize approaches to compete for user attention against messages that promote unhealthy behaviors from diverse product marketers [6].

As social media has evolved, so have the marketing techniques used by businesses to push their products and ideas on the population. Social marketing of public health goods is also finding its place on this environment, with social media becoming widely recognized as “an unprecedented opportunity...to deliver socially influential online behavior change interventions” [7]. Social marketers have adapted commercial marketing techniques to promote the adoption and maintenance of health behaviors (eg, identify objectives of targeted behavior changes and tailor messages for target audiences) [6,8]. Furthermore, there is a growing body of literature that such online health interventions can increase knowledge and understanding of health topics, including smoking cessation, diet, and exercise [7,9-11].

Specifically, social media has been identified as an opportunity for a potentially cost-effective approach to improve breastfeeding outcomes relative to traditional social marketing, yet research for social marketing for breastfeeding promotion is needed to identify best practices and approaches [8,12,13]. To date, there are no published studies on efficient and effective approaches to disseminate breastfeeding information via social media. Given the significance of social support as a determinant of breastfeeding [13], a social media–based breastfeeding campaign that taps into social networks and connections to disseminate the campaign messages may improve the success and acceptability of a campaign among the target population.

In social media marketing, consumer-to-consumer interaction and word-of-mouth dissemination of information is widely used in the form of influencers [14]. Social network targeting, an application of social network analysis, has been tested in Honduras to evaluate impacts on adoption of chlorine tablets and multivitamins use [15]. Through this approach, social network analysis indicators are used to identify socially influential individuals to spread an intervention, idea, or product. This approach has the potential to optimize the dissemination of breastfeeding information in a social media–based campaign through the social connections and interactions between socially influential individuals and individuals previously unreached and uninterested by the campaign messages.

Alternatively, social media platforms have established mechanisms for businesses to pay for advertisements to appear on the news feed of targeted consumer groups. These paid advertisements have been effective for business, especially when paired with creative marketing, and as a result are considered essential in business social media marketing plans [16]. This is due in part to the social media platform’s algorithms that limit the amount of posts from business pages that appear on consumer’s feeds, unless the business pays. This can be achieved through advertising specific posts or advertising the full business page or account. Overall, numerous strategies have been considered and published with regard to effective social media marketing, which generally position social media within the context of broader business or product marketing strategies, such as brand awareness [17-19].

Between 2008 and 2014, the rate of children under 6 months of age who were exclusively breastfed in Ghana declined from 63% to 52% [20,21]. In response, Ghana’s Becoming Breastfeeding Friendly (BBF) Initiative committee (led by the University of Ghana) identified key gaps in the national breastfeeding environment and recommended social media as a platform on which specific gaps could be filled [22]. Social media penetration has rapidly risen in Ghana in recent years to reach 32% among adults in 2017 and 43% among 18- to 36-year-old, with 2 of the most popular platforms in the country being Facebook and Twitter [23]. On the basis of the BBF Initiative recommendations and rising popularity of social media, the Breastfeed4Ghana social media–based campaign was designed and implemented with the aim of disseminating messages on breastfeeding protection, promotion, and support on Facebook and Twitter [24]. The campaign targeted the broad population of Ghanaian adults, given that campaign messages included supporting women to breastfeeding and protecting maternity leave legislation, which are relevant to the general public.

Therefore, the primary aim of this study is to evaluate the impact of different dissemination paths on exposure and engagement with campaign material and to examine the relationship of acceptability of campaign material with material performance.

## Methods

### Design

We implemented a 6-month long Facebook (Menlo Park, CA, US) and Twitter (San Francisco, CA, US) campaign that targeted the protection, promotion, and support of breastfeeding in Ghana, on the basis of evidence from recommendations from the BBF Initiative in Ghana [22]. The methods of the campaign design have been previously published [24]. In brief, a total of 60 core campaign materials, each consisting of a brief message and a corresponding photograph, were iteratively developed via 6 focus group discussions among Ghanaian mothers to gain input on message and image acceptability, understandability, and alignment of the message and image. Materials were also reviewed by various content and technical experts in infant and young child feeding and were approved by Ghana’s Food and Drug Authority. Materials represented 3 campaign themes: (1) promote correct and complete information about breastfeeding; (2) support women to breastfeed anytime and anywhere; and (3) protect working women’s right to breastfeed. These materials were disseminated initially on Facebook and Twitter over a 12-week period, during which 5 materials were posted simultaneously on both platforms at the same date and time each week; 40 of these core campaign materials were chosen on the basis of their engagement performance (ie, materials that performed better, as described below) for redissemination during a subsequent 8-week period. Similar to the 12-week initial dissemination period, 5 materials were posted on both platforms at the same date and time each week. In addition to a Facebook page and Twitter page, a Facebook profile for the campaign coordinator was established before the campaign was launched to engage with those interested in the campaign further.

Campaign performance for platforms (ie, Facebook and Twitter pages) and individual material performance (ie, Facebook posts and tweets) were monitored using data extracted from Facebook Insights and Twitter Analytics and entered into a Microsoft Access database (Microsoft Corp, Redmond, WA, US). Platform data were collected weekly and included the number of followers and likes on both Facebook and Twitter; and reach, engagements, views, and follower and engagement demographics by age, sex, and country on Facebook. Data on the campaign posts, such as the core campaign materials, were extracted at 3 timepoints: 1 day, 1 week, and 2 weeks after the material was posted. These data included material impressions or reach, likes, share or retweets, and comments or replies. Materials were selected for redissemination in the test phase based on a composite indicator of the amplification and applause rates per 100 followers for each Facebook and Twitter at the 1 day time point. Thus, materials were ranked from highest to lowest performance, by theme, and the top performing 40 campaign materials (13 or 14 per theme) were selected for redissemination across an 8-week period.

**Figure 1:**
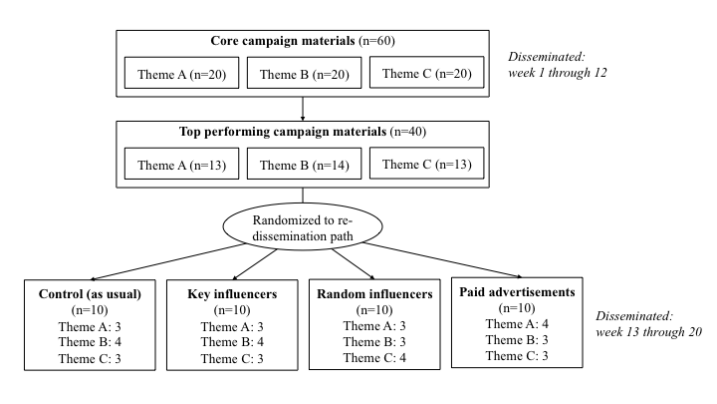
Study design for testing dissemination paths via Facebook and Tweeter. Theme A: Promote correct and complete information about breastfeeding; Theme B: Support women to breastfeeding anytime, anywhere; Theme C: Protect working women’s right to breastfeed.

Stratified by theme, materials were randomized to 1 of 4 redissemination paths: (1) posted as usual (ie, control group); (2) shared by 6 key influencers on Facebook and 6 key influencers on Twitter after being posted; (3) shared by 6 random influencers on Facebook and 6 random influencers on Twitter after being posted; or (4) paid advertisement with US $6 on each Facebook and Twitter after being posted (Figure 1). Each dissemination pathway was assigned a corresponding number (1 through 4), and using a random number generator without replacement, each theme was randomly assigned 1 dissemination pathway (themes A and C) or 2 dissemination pathways (theme B) that would be assigned 4 materials, while the other pathways would be assigned 3 materials, to achieve balance across dissemination pathways by theme. Within each theme, materials were listed in descending order by performance score, and using a random number generator (1 through 4), each material was randomly assigned to a dissemination pathway. Once a pathway reached the number of materials to be assigned (ie, 3 or 4 materials), no more materials were assigned to that pathway.

### Influencer Selection

To select the influencers, publicly available details (ie, number of friends or followers, number of friends or followers in common with the campaign, recent activity, and country of residence) from Facebook and Twitter user profiles were extracted from the respective platform among *friends* from the Breastfeed4Ghana campaign coordinator’s Facebook profile and followers from the Twitter page. The sampling frame for the influencers was the Breastfeed4Ghana Facebook *friends* and Twitter followers, excluding individuals who did not reside in Ghana. The size of an individual’s reach was calculated based on the size of their network that did not overlap with the campaign’s network based on the number of friend or follower that the individual had that were *not* in common with the campaign (ie, number of followers or friends *minus* the number of follower or friends in common with the campaign). This indicator of reach was used as an approximation of the significance of each individual in these social networks [25,26].

The selection of key influencers focused on influencer reach and network ties, and therefore, the previous level of involvement with the campaign was not a selection criterion, although all influencers were at least following the campaign. Those with the highest reach were vetted by the campaign coordinator to make sure that they did not have inappropriate content (eg, pornography, violence, extremist behavior, or other offensive content) on their social media page and were then invited to be a key influencer for the campaign. It was explained to potential influencers that as an influencer they would be asked to share or retweet 1 to 2 campaign posts per week for 8 weeks and would receive a small incentive for their time of 80 Ghana cedis (~ US $20 or US $2.50 per week) for sharing the posts. This was repeated until a total of 6 key influencers on each of Facebook and Twitter were selected. To achieve this sample of 6 key influencers per platform, a total of 19 individuals were approached on Facebook and 9 on Twitter. To select random influencer, the remaining individuals in the sampling frame *after* the key influencers had been selected were numbered. We randomly selected individuals from this list using a random number generator. Selected individuals were vetted by the campaign coordinator to ensure they did not have inappropriate content on their page and invited to be a campaign influencer. This was repeated until 6 *randomly* selected influencers were confirmed for each Facebook and Twitter. To reach 6 randomly selected influencers on each platform, we invited 13 individuals on Facebook and 14 individuals on Twitter. Influencers were sent weekly instructions to share or retweet specific posts on Facebook and Twitter, respectively.

### Paid Advertisements

Scheduled, targeted advertisements for the selected campaign materials were achieved through business accounts on both Facebook and Twitter, equating to US $6 per platform for each material randomly assigned to the paid advertisement dissemination arm. On Facebook, advertisements targeted 18 to 49 years old women residing in Ghana and were conducted in the form of a post *boost*. The objective of the advertisement was set to *post engagement*, which aims to gain more views and engagement, such as *likes* and *shares*, for the post [27].

For Twitter, acquiring a business account in Ghana required going through a third-party company, which required meeting a minimum quarterly advertising budget. Similar to Facebook, advertisements targeted individuals 18 to 49 years old residing in Ghana. For both Facebook and Twitter, the paid advertisement was schedule along with the posts being scheduled, which were generally scheduled at least 1 week before the post date.

### Campaign Material Acceptability Survey

Campaign material acceptability was assessed via an online survey conducted over 3 timepoints. Each survey timepoint corresponded with the completion of the initial dissemination of campaign materials from 1 of the 3 campaign themes (ie, dissemination during the initial 12-week campaign period) and asked about the core campaign materials from that particular theme. Survey participants were a convenient sample of women ≥ 18 years of age residing in Ghana. The survey was promoted through an advertisement post on the campaign’s Facebook and Twitter pages and completed through Qualtrics (Provo, UT, USA). Materials presented in the surveys were chosen on the basis of material performance to represent the bottom, median, and top performance within the respective campaign theme, with performance on the basis of the number of *likes* and *share* on Facebook and *likes* and *retweets* on Twitter. For each material, participants were asked questions regarding their understanding and acceptability of the image, the message, and the overall material; 10 questions were asked, based on a 5-point Likert scale ranging from *strongly disagree* to *strongly agree* (5 questions regarding the image and 5 regarding the message); 3 questions asked about the overall rating on a 5-point Likert scale (from *very bad* to *very good*) of each of the image, message, and material, respectively. Performance was calculated as a composite indicator of the amplification and applause rates per 100 followers for each Facebook and Twitter at the 1-day time point. A total of 9 campaign materials were evaluated (Multimedia Appendix 1).

### Variable Generation

All data were imported into Stata 14.1 (Stata Corp, College Station, TX, USA) for cleaning and analysis.

Key performance indicators (KPIs) for platform and material exposure and engagement were developed from available data and based on Neiger et al 2012 definition of KPIs [28].

Platform Engagement was defined as the number of followers on each Facebook and Twitter. For this analysis, this KPI was used to adjust material KPIs by follower based at the time the material was posted, to make them comparable across time and platform. As reported elsewhere, the campaign started with 3061 Facebook followers and 27 Twitter followers and ended with 4096 Facebook followers and 736 Twitter followers [24].

Material Exposure was defined as reach on Facebook, which is the number of unique people who saw the material; and as impressions on Twitter, which is the number of times the material appeared on a Twitter timeline. These values were converted into rates per 100 followers on the respective platform at the time of the material posting to make them comparable across time and platform. These rates were used at the primary KPI for material exposure on each Facebook and Twitter.

Material Engagement comprises 3 subindicators: applause, amplification, and conversation. Applause was defined as the number of likes on each Facebook and Twitter; amplification was defined as the number of shares on Facebook and retweets on Twitter; and conversation was the number of comments on Facebook and replies on Twitter. Each of these subindicators by platform were converted to a rate per 100 followers on the respective platform at the time of the material posting (ie, (subindicator ÷ number of follower) × 100). Applause and amplification rates per 100 followers from all material posts and timepoints (n=300) for each Facebook and Twitter were standardized (μ=0; σ=1), and summed by platform and collectively to generate 3 material engagement scores for each material: total engagement score, Facebook engagement score, and Twitter engagement score.

In the content analysis survey, 13 statements examined the material image, message, and overall acceptability. The 5-point-Likert response options were collapsed to emphasize *positive* response options, thus agreement included *extremely agree* and *agree*, and not in agreement included *neutral*, *disagree*, and *extremely disagree* for analysis. For negative statements, such as *the picture is confusing*, the responses were collapsed to emphasize disagreement (*extremely disagree* and *disagree*); and for the overall rating questions, responses were collapsed to good (*very good* and *good*) and not good (*neutral*, *bad*, and *very bad*).

### Statistical Analysis

To determine the impact of dissemination path on material performance, a series of difference-in-difference models that accounted for material performance at baseline were run for each of the following material KPI: Facebook exposure, Twitter exposure, total engagement score; Facebook engagement score, and Twitter engagement score. Given the difference in the indicator for exposure on Facebook versus Twitter, a total engagement score was not tested.

To determine characteristics of campaign materials that related to higher material performance, content survey data was pooled across the 3 timepoints, and material performance (low, middle, and high) was examined in relation to material acceptability defined as agreement with each of 6 acceptability statements, disagreement with 4 negative statements, and rating the image, message, and material as *good* in the respective 3 questions. Logistic regression models, controlling for the survey taken (1, 2, or 3), and the respondent examined the odds of binary material acceptability across low, middle, and high material performance.

### Ethical Approval

This study was approved by the Yale University Institutional review board and the review board for Ghana University hosted by the Noguchi Institute. Influencers and content survey participants provided their electronic consent to participate in the study before their respective participation.

## Results

### Overall Performance

During the 12-week long baseline period, the 60 core campaign materials received an average (SD) exposure of 1178 (670) on Facebook and 1071 (905) on Twitter (Table 1). On both Facebook and Twitter, the majority of engagement was seen in applause, followed by amplification. On both platforms, minimal conversation was observed. All measures of material exposure and engagement were larger in absolute values on Facebook compared with Twitter, and larger in rates per 100 followers on Twitter than on Facebook, with the exception of conversation.

### Dissemination Paths

The top 40 performing core campaign materials were selected for the dissemination test period, with baseline characteristics summarized in Table 1. Among these 40 materials, there were no statistically significant differences across the 4 arms in baseline exposure, applause, amplification, and conversation per 100 followers at *P*<.05.

**Figure 2:**
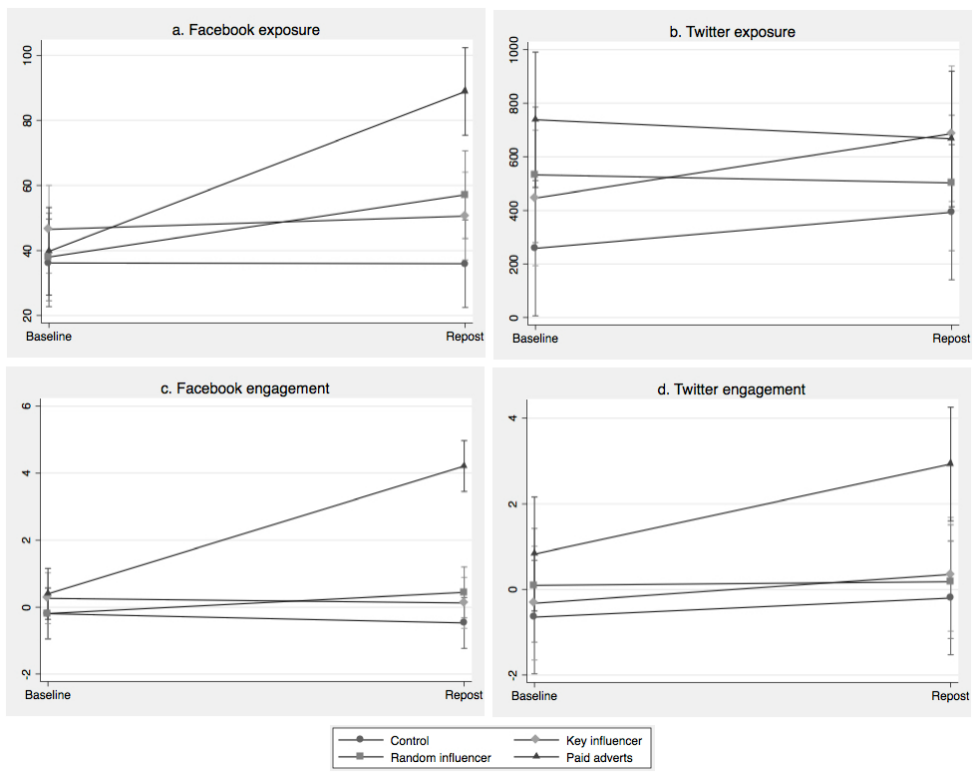
Difference-in-difference models for material exposure on Facebook (a) and Twitter (b), and material engagement on Facebook (c) and Twitter (d) across dissemination path arms (95% CIs).

KPI for exposure on Facebook and all engagement indicators tending to increase from baseline to the test period, though these increases were only significant in the paid advertisement arm (Table 2). When these differences were examined across arms, paid advertisements yielded significantly higher exposure and engagement on Facebook, compared with the control group (Figure 2). Specifically, Facebook exposure increased by 124% in the paid advertisement group (from 39.76 impressions per 100 followers to 88.88), compared with a decrease of 1% in the control arm (36.20 impressions per 100 followers to 35.96; *P*<.01). Similarly, the Facebook engagement score in the paid advertisement group increased by 953% (0.40 engagement per 100 followers to 4.21), compared with an increase of 147% in the control arm (0.19 engagement per 100 followers to 0.47; *P*<.01). There were no statistically significant differences between the 2 influencer arms and the control arm.

All 40 posts were disseminated via scheduled posts on both Facebook and Twitter at baseline and during the test period. Among the 10 materials randomly assigned to the paid advertisement arm, all had the paid advertisements directed at adults (18-49 years) in Ghana scheduled alongside the post schedule, as planned. Among the 20 materials that were randomized to the key influencers (n=10 materials) and random influencer (n=10 materials) arms, influencers were requested to share the material within 48 hours; 2 of the 6 key influencers on Facebook did not share all 10 materials that were requested and 1 of the 6 key influencers on Twitter did not share all the materials requested. All of the random influencers on Facebook (n=6) and Twitter (n=6) shared all 10 materials requested.

**Table 1:** Definitions and summary of baseline performance indicators for core campaign materials on Facebook and Twitter, representing all 60 core campaign materials and the subsample of 40. Table is based on data collected 2 weeks after the material was posted.

Performance indicator and platform			Definition	Baseline (n=60)		Baseline (n=40)^a^	
				Mean (SD)	Mean (SD) per 100 followers	Mean (SD)	Mean (SD) per 100 followers
**Exposure**							
	Facebook		Reach^b^	1178 (670)	33.12 (19.12)	1425 (655)	40.12 (18.84)
	Twitter		Impressions^c^	1071 (905)	375.98 (396.34)	1345 (951)	494.13 (433.08)
**Engagement**							
	**Applause**						
		Facebook	Likes	56.03 (34.01)	1.59 (0.99)	67.13 (36.08)	1.90 (1.06)
		Twitter	Likes	4.28 (2.99)	1.63 (2.14)	5.23 (2.97)	2.12 (2.46)
	**Amplification**						
		Facebook	Shares	9.37 (5.11)	0.26 (0.15)	11.45 (4.77)	0.32 (0.14)
		Twitter	Retweets	2.23 (2.07)	0.81 (1.14)	2.83 (2.07)	1.07 (1.29)
	**Conversation**						
		Facebook	Comments	1.33 (2.66)	0.038 (0.076)	1.9 (3.08)	0.054 (0.088)
		Twitter	Replies	0.02 (0.13)	0.004 (0.033)	0.03 (0.16)	0.006 (0.041)

^a^Top and middle performing material based on engagement (sum of applause, amplification, and conversation).

^b^Reach is unique people saw content on Facebook.

^c^Impressions refers to times it appeared on a Twitter timeline.

**Table 2:** Material performance at baseline and repost (test period) by dissemination path arms.

Key performance indicators		Control, mean (SD)	Key influencers, mean (SD)		Random influencers, mean (SD)		Paid advertisements, mean (SD)		*P* value^a^
		RP^b^	BL^c^	RP	BL	RP	BL	RP	
**Exposure^d^**									
	Facebook	35.96 (8.64)	46.51 (31.43)	50.61 (26.87)	38.01 (13.36)	57.19 (35.41)	39.76 (13.87)	88.88 (11.79)^e^	<.001
	Twitter	393.57 (312.14)	446.43 (300.58)	686.34 (358.34)	533.02 (444.02)	502.49 (278.32)	738.3 (577.61)	666.85 (548.28)	.58
**Engagement^f^**									
	Combined	−0.67 (0.59)	−0.05 (1.32)	0.48 (1.13)	−0.09 (1.62)	0.63 (1.43)	1.23 (2.49)	7.14 (4.18)^e^	<.001
	Facebook	−0.47 (0.51)	0.27 (1.43)	0.13 (1.02)	−0.19 (0.81)	0.44 (1.57)	0.40 (1.11)	4.21 (1.81)^e^	<.001
	Twitter	−0.20 (0.44)	−0.32 (0.41)	0.35 (0.51)	0.10 (1.34)	0.18 (0.40)	0.83 (2.54)	2.93 (5.10)	.36

^a^P value for dissemination arm by repost interaction in the difference-in-difference model.

^b^RP: repost time point.

^c^BL: baseline.

^d^Defined as reach per 100 followers on Facebook and impressions per 100 followers on Twitter.

^e^*P*<.01 for key performance indicator between baseline and repost.

^f^Defined as the sum of the standardized applause and amplifications rates per 100 followers for both platforms combined, and individually.

### Performance and Acceptability

A total of 44 female participants completed the content survey (Table 3). The majority of participants were based in Greater Accra (68%, 30/44), married (43%, 19/44), employed (77%, 34/44), and highly educated (86%, 38/44). All participants had access to their own smartphone and 73% (32/44) and 32% (14/44) daily accessed Facebook and Twitter, respectively.

Respondents reported the campaign materials to be acceptable, with over 75% agreement (or disagreement to negative statements) for 12 out of 13 acceptability statements across all materials (Table 4). Agreement ranged from 64% with the statement *I like this picture* to 91% with the statement *this message is informative.* The odds of acceptability of the material image were significantly greater among the high-performance material compared with the low-performance materials, based on 3 out of the 5 acceptability statements at *P*<.05 (Table 4). Similarly, the odds of acceptability of the material message was significantly greater among the middle-performance material compared with the low-performance materials, based on 2 out of the 5 acceptability statements. The odds of overall acceptability, based on rating the image and material as *good*, was significantly greater among both the middle- and high-performance material compared with the low-performance material; and the odds of rating the message as *good* was significantly greater among the middle-performance material compared with the low-performance material.

**Table 3:** Survey participant characteristics (N=44).

Characteristics		Value
Age (years), mean (SD)		28.61 (4.20)
Based in Greater Accra, n (%)		30 (68)
Married, n (%)		19 (43)
Employed, n (%)		34 (77)
Education: bachelor’s or higher, n (%)		38 (86)
Had children, n (%)		18 (41)
**Daily access in the past week to:**		
	Internet, data, and Wi-Fi, n (%)	37 (84)
	Facebook, n (%)	32 (73)
	Twitter, n (%)	14 (32)
Access to own smartphone, n (%)		44 (100)

**Table 4:** Material acceptability across material performance levels.

Statements and rating		Prevalence (SE) of *agreement* with statement overall, and by material performance (N=44)				Odds ratio (SE): measure of material acceptability by material performance^a^		
		All	Low	Middle	High	Low	Middle	High
**Image**								
	This picture promotes breastfeeding	79.55 (3.51)	70.45 (6.88)	79.55 (6.08)	88.64 (4.78)	Reference	1.69 (0.90)	3.52 (2.08)^b^
	The picture is informative	78.03 (3.60)	68.18 (7.02)	77.27 (6.32)	88.64 (4.78)	Reference	1.69 (0.95)	4.13 (2.31)^b^
	This picture is confusing^c^	81.06 (3.41)	75.00 (6.53)	75.00 (6.53)	93.18 (3.80)	Reference	1	4.59 (2.85)^b^
	I like this picture	64.39 (4.17)	59.09 (7.41)	61.36 (7.34)	72.73 (6.71)	Reference	1.10 (0.41)	1.86 (0.72)
	This picture is misleading/dishonest^c^	87.88 (2.84)	86.36 (5.17)	88.64 (4.78)	88.64 (4.78)	Reference	1.23 (0.78)	1.23 (0.69)
**Message**								
	This message promotes breastfeeding	87.12 (2.92)	79.55 (6.08)	95.45 (3.14)	86.36 (5.17)	Reference	5.50 (4.79)^b^	1.64 (0.92)
	The message is informative	90.91 (2.50)	81.82 (5.81)	100.00 (0.0)	90.91 (4.33)	Reference	—^d^	2.28 (1.32)
	This message is confusing^c^	86.36 (2.99)	86.36 (5.17)	88.64 (4.78)	84.09 (5.51)	Reference	1.23 (0.87)	0.83 (0.46)
	I like this message	80.30 (3.46)	72.73 (6.71)	88.64 (4.78)	79.55 (6.08)	Reference	2.99 (1.54)^b^	1.47 (0.50)
	This message is misleading/dishonest^c^	87.79 (2.86)	84.09 (5.51)	90.91 (4.33)	88.37 (4.89)	Reference	1.90 (1.09)	1.44 (0.69)
**Overall rating as good^e^**								
	Image	76.52 (3.69)	61.36 (7.34)	79.55 (6.08)	88.64 (4.78)	Reference	2.50 (1.23)^b^	5.07 (2.91)^f^
	Message	87.88 (2.84)	79.55 (6.08)	95.45 (3.14)	88.64 (4.78)	Reference	5.54 (4.32)^b^	2.03 (1.00)
	Material	78.03 (3.60)	61.36 (7.34)	86.36 (5.17)	86.36 (5.17)	Reference	4.15 (1.84)^f^	4.15 (2.05)^f^

^a^Odds ratio for logistic regression models adjusted for content survey and respondent.

^b^*P*<.05.

^c^Prevalence represents disagreement with statement.

^d^Could not calculate Odds ratio because prevalence in middle performing group was 100%.

^e^Prevalence represents rating as *good*.

^f^*P*<.01.

## Discussion

### Principal Findings

Breastfeed4Ghana core campaign materials achieved higher exposure and engagement on Facebook than on Twitter, and higher exposure and engagement rates per 100 followers on Twitter than on Facebook, because of the relatively small number of followers on Twitter. In this study, paid advertisements significantly increased material exposure and engagement on Facebook. Although neither influencer type had a significant impact on material performance on Facebook, there was a trend in higher engagement and exposure as a result of random influencers, compared with baseline. Conversely, there were no significant differences across the 4 dissemination paths on Twitter (ie, control, key influencers, random influencers, and paid advertisements), and it is important to consider the variations in purposes of different social media platforms and how they are used and how to consider this variation as part of the *social media ecosystem* [2,17].

A total of US $6 per material per platform was allocated to advertisement for each material in the paid advertisement arm. Accounting for the airtime incentives provided to influencers across the test period, each material in the influencer arm *cost* US $12 per platform for promotion. Furthermore, there was more personnel time required to share the materials with the influencers, follow-up with influencers, and provide the incentives to influencers. Influencer management accounted for approximately 90 min per week of the campaign coordinators times, compared with approximately 10 min/week to manage the advertisements. Thus, the findings from this study indicate that paid post advertisements, through Facebook’s business account, was not only most effective in increasing material performance but also at a lower cost than our model for either key or random influencers.

Word-of-mouth is a recognized marketing approach for the expansion of a product or idea [29], and the use of influencers is a way to amplify the word-of-mouth on social media. We employed a technique of microinfluencers, who are individuals with typically more than 10,000 followers. Microinfluencers are contrasted with macroinfluencers, who are individuals with much larger followings and include well recognized celebrities [30]. Both types of influencers have been used across different social media platform to promote products or ideas and microinfluencers have been touted as able to achieve more engagement than the macroinfluencers and at a lower cost [31].

There is no prescribed way to recruit, select, and manage influencers, as Keller and Fay describe various case studies in their business marketing report, and influencers generally can impact various outcomes such as message amplification and product sales [14]. In this study, it was surprising that neither type of influencer yielded higher engagement or exposure with the campaign posts. It is possible that a greater number of influencers would be required to achieve such impacts on performance. It is also possible that the target population of Ghanaian adults was too broad, and a more focused target population of new mothers in Ghana would have been more effective.

Our key influencers were selected with consideration for social network targeting, and also aimed to examine the difference in selecting key influencers (ie, social network targeting) as is done often with micro- and macroinfluencers versus randomly selected influencers. Similar to findings within a community health program in Honduras, between social network targeting and randomly selecting influencers, there were not significantly different outcomes; however, in Honduras, both social network targeting (akin to macroinfluencers) and random selection yielded significantly higher adoption of the intervention than the control group [15]. The lack of a difference on both Facebook and Twitter found between targeted and randomly selected influencers suggest that it is not necessary to expend resources to select *highly influential* individuals. As well, in this study, the randomly selected influencers were more adherent to sharing posts compared with the key influencers. Customer-to-customer interactions, such as those prescribed to influencers, has further been modeled in the marketing literature to be able to start a chain effect among consumers, with lasting impacts [32], which may make it superior to a paid advertisement approach in some context. Such superiority of influencers to paid advertising was achieved in a Twitter-based skin cancer prevention campaign [33]. Yet, findings from this study are discordant with the Ireland skin cancer prevention campaign, in that the influencer promotion did not impact post performance, which may be the result of contextual differences in topic and target audience.

In our examination of domains of material acceptability and material performance, overall acceptability of the image, message, and material were associated with performance in terms of engagement. Most notable was that highest performing materials were those that had images viewed as (1) promoting breastfeeding; (2) informative; and (3) *not* confusing. Research which aims to shed light on social media consumer behavior and interaction with content provides insights into findings from this study. Indeed, Berger postulated a framework for the drivers of viral content: social currency, triggers, emotion, public, practical information, and stories [34]. Yuki expanded on this work by evaluating the 2000 most and least shared Facebook posts by various brands between 2013 and 2014 using an online survey among 10,083 individuals in the United States [35]. Findings from this study suggest that higher performing posts were viewed as informative, which aligns with the viral posts surveyed in Yuki’s study and with Berger’s framework. High-performing materials were also those with images viewed as promoting breastfeeding, which may provide social currency—in an environment where breastfeeding is typically viewed positively and promoting breastfeeding could be viewed as looking good or intelligent.

While this campaign generated 60 core campaign materials that were disseminated 1 to 2 times during the active campaign period, and as a result varied the images so as not to be too repetitive (a feedback from our formative material development work). Despite the variety of images presented in the campaign materials, the highest performing material for each theme was that of a woman breastfeeding. This may suggest that such variety (ie, 60 unique materials) was not necessary, and generating images most aligned with the campaign focus and message will yield the greatest engagement and acceptability among the target population. As such, other social marketing campaigns have generated a small number of messages and materials that are widely and repeatedly disseminated [36].

This study also reports on KPIs of campaign materials. In public health, return on investment is not always a useful indicator, and Neiger et al summarized KPIs and evaluation metrics for health promotion on social media [28]. Although their work provides an important list of possible KPIs and corresponding metrics, adoption and reporting of such indicators in the health promotion field is not widespread. This is an important *data gap*, both in terms of reporting and consideration for consistency in metrics. Although different campaigns and studies will have different goals that should drive indicators of performance or success, work by Neiger and metrics reported in this study can provide a guide for other campaigns.

### Limitations

To our knowledge, similar studies have not been published. However, paid advertisements, as well as paid influencers, are widespread social marketing techniques used across businesses and industry. It is important to recognize the limitation in lack of comparable studies and results for us to consider. These results also reflect a short-term study and small number of influencers. Therefore, it is possible that in a study of longer duration with a higher number of influencers could yield different results. The lack of differences across dissemination path arms on Twitter could be the result of a small follower based on that platform, as well as a significant increase in follower (approximately doubled) right before the test period of the study. Finally, we would like to acknowledge that these results come from a breastfeeding social media–based campaign that targeted the population of Ghana. There are variations in how social media is used in different contexts across geographic space, demographics, and time, and therefore, generalizability of these results beyond the context should be done with caution. Similarly, for context, it is important to note the dates of from this campaign (March to September 2018). Social media platforms are continually updating their business platforms, advertising option, and algorithms for what is viewed on user’s news feeds, and therefore, comparability of the findings from this study may be limited based on how the landscape of social media changed and evolves with time.

### Conclusions

Paid advertisements are an effective mechanism to increase exposure and engagement of campaign posts on Facebook, achieved at a low cost. Although influencers are used in marketing and are generally considered effective at increasing consumer engagement or sales, microinfluencers were not effective at increasing exposure and engagement in this study. Furthermore, the use of influencers to promote materials required a greater financial cost compared with paid advertisements in our study.

For social marketing, there are challenges with how to compete against product advertisers with bigger budgets and more ways to reach consumers (eg, infant formula companies) [6]. As social media marketing campaigns continue to rise in popularity for health behavior research, common metrics for evaluating campaign performance, such as platform and material performance, and how campaign outcomes and impacts are reported should be used. This study contributes to a small, but growing, body of literature on KPIs in social media health behavior and promotion campaigns.

## Appendix

Multimedia Appendix 1 Materials tested in the content survey.

## Abbreviations

BBF: becoming breastfeeding friendly
KPI: key performance indicator
