# Supplementary material for: Four Dissemination Pathways for a Social Media–Based Breastfeeding Campaign: Evaluation of the Impact on Key Performance Indicators
Source: JMIR Nurs. 2019 Sep 26;2(1):e14589. doi: 10.2196/14589 (PMC8293701; doi:10.2196/14589)

Appendix 1. Materials tested in the content survey

Survey 1 – Low (a), Middle (b) and High (c) performing material from Theme A: Promote correct and complete information about breastfeeding

a)

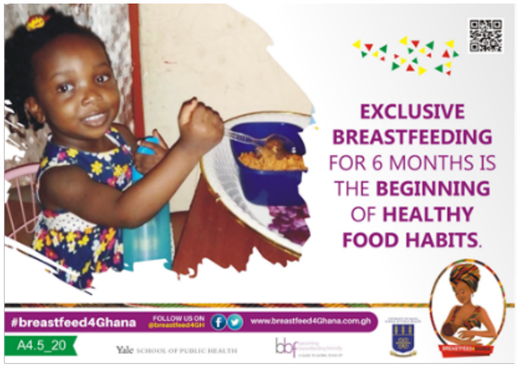

b)

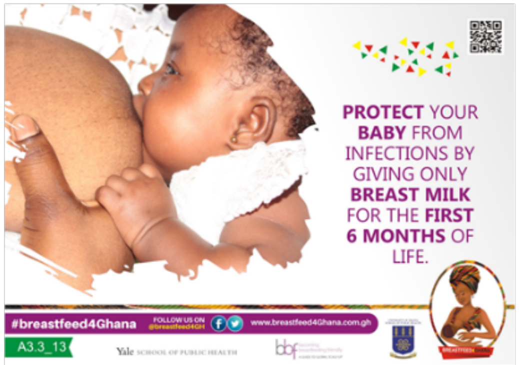

c)

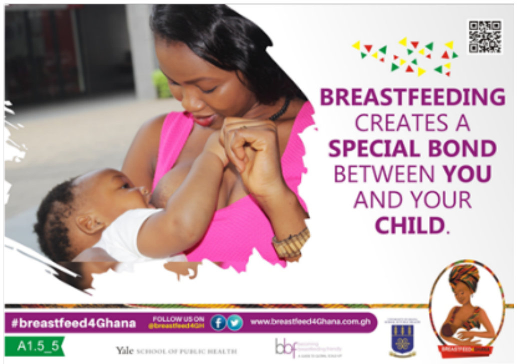

Survey 2 – Low (a), Middle (b) and High (c) performing material from Theme B: Support women to breastfeeding anytime, anywhere

a)

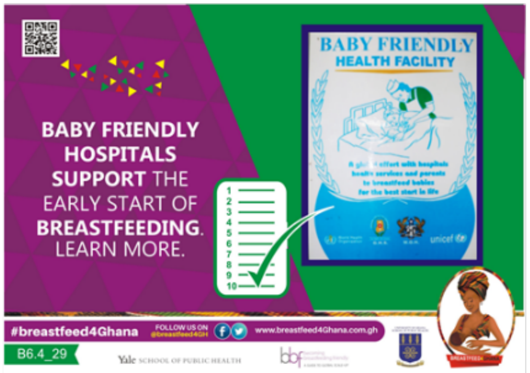

b)

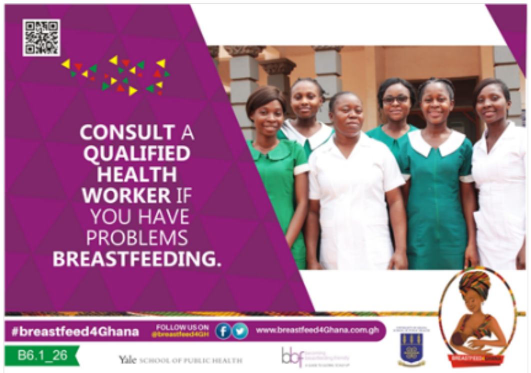

c)

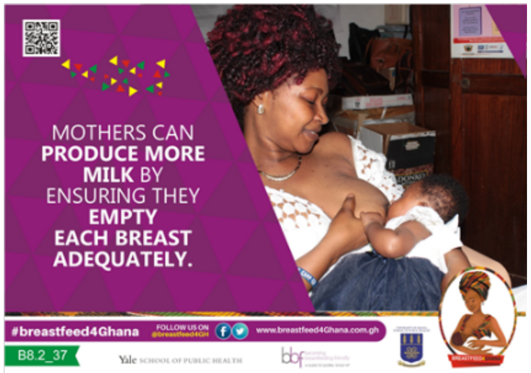

Survey 3 – Low (a), Middle (b) and High (c) performing material from Theme C: Protect working women’s right to breastfeed

a)

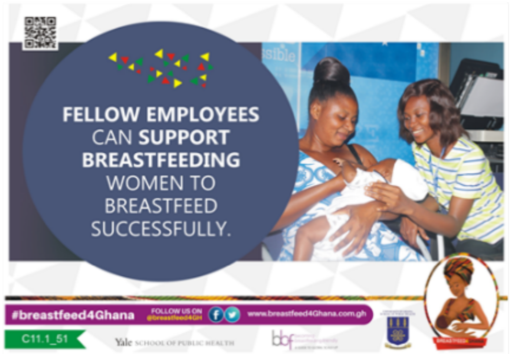

b)

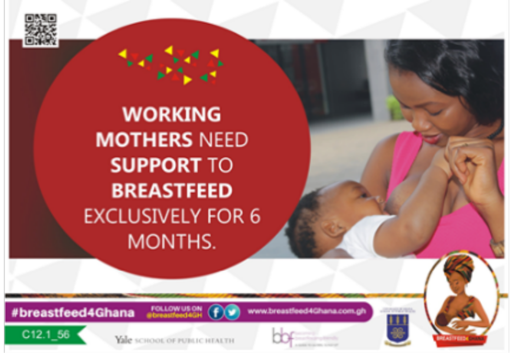

c)

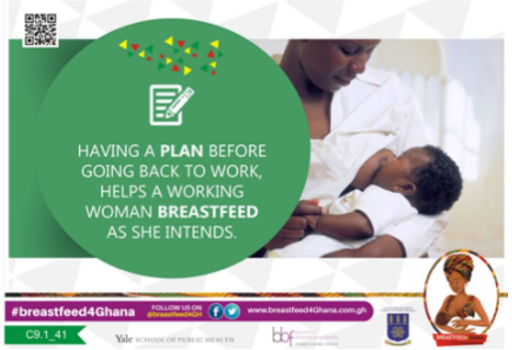

Supplement: Multimedia Appendix 1 [file nursing_v2i1e14589_app1.pdf]
